# Urban green roofs provide habitat for migrating and breeding birds and their arthropod prey

**DOI:** 10.1371/journal.pone.0202298

**Published:** 2018-08-29

**Authors:** Dustin R. Partridge, J. Alan Clark

**Affiliations:** Department of Biological Sciences, Fordham University, Bronx, NY, United States of America; University of Southern California, UNITED STATES

## Abstract

The world is rapidly urbanizing, and many previously biodiverse areas are now mostly composed of impervious surface. This loss of natural habitat causes local bird communities to become dominated by urban dweller and urban utilizer species and reduces the amount of habitat available for migrating and breeding birds. Green roofs can increase green space in urban landscapes, potentially providing new habitat for wildlife. We surveyed birds and arthropods, an important food source for birds, on green roofs and nearby comparable conventional (non-green) roofs in New York City during spring migration and summer breeding seasons. We predicted that green roofs would have a greater abundance and richness of both birds and arthropods than conventional roofs during both migration and the breeding season for birds. Furthermore, we predicted we would find more urban avoider and urban utilizer bird species on green roofs than conventional roofs. We found that both birds and arthropods were more abundant and rich on green roofs than conventional roofs. In addition, green roofs hosted more urban avoider and utilizer bird species than conventional roofs. Our study shows that birds use green roofs as stopover habitat during migration and as foraging habitat during the breeding season. Establishing green roofs in urban landscapes increases the amount of habitat available for migrating and breeding birds and can partially mitigate the loss of habitat due to increasing urbanization.

## Introduction

The majority of humans now reside in urban landscapes [1], and many of the most dense human populations are located near, or in, areas with high faunal richness [2, 3]. The co-occurrence of high human density and high species richness is not due to humans having a positive influence on species richness [4]. Rather, areas of high human density and high biodiversity typically occur in areas with increased productivity, high habitat heterogeneity, and easily accessible resources [2, 5, 6]. As the human population continues to grow, urban landscapes are expected to expand as well [1]. These expanding urban landscapes will encroach further into more biodiverse habitats causing disruptive change to both invertebrate [7–9] and vertebrate populations [10, 11].

Expanding urbanization is likely to have large impacts on bird communities [12]. For example, urbanization increases bird mortality due to collisions with human structures and increases predation, primarily by free-ranging domestic cats [12–14]. Urbanization also results in the destruction of natural habitats, altering bird community composition as some species benefit from human-dominated landscapes while other species are affected negatively [15–20].

This differential response by bird species to urbanization depends, in part, on species’ relative ability to tolerate and take advantage of urban landscapes. Most bird species can be classified as urban dwellers, urban utilizers, or urban avoiders [16, 19, 21]. Urban dwellers [21], also known as urban exploiters [16, 19], are species that thrive in urban landscapes independent of natural areas (i.e., green spaces such as remnant or restored habitats [21]) and typically have highly flexible diets, are behaviorally flexible, or have co-evolved with humans [21, 22]. Urban utilizers [21], also known as urban adapters [16, 19], are species that can use urban landscapes but do not generally benefit from them and are typically native species that still rely primarily on more natural areas (i.e., areas minimally modified for human use [16, 19, 21]). Urban avoiders are species most negatively impacted by urbanization and are typically native and specialist species that only use non-urban habitat or are not usually found in urban landscapes [16, 19, 21]. However, urban avoiders may nonetheless be found in urban landscapes if such landscapes contain patches of green space containing natural areas [21, 23–27]. Urban dwellers and urban utilizers are commonly omnivores or granivores while urban avoiders tend to be insectivores [16, 19, 22, 28].

Urban green spaces are used by bird species with differing life histories, including birds that are migratory and birds that are year round residents [27, 29–32]. Some migratory birds end their migration in urban landscapes where they breed; others use urban landscapes as stopover sites before heading on to their final breeding grounds. Regardless of final breeding location, the majority of migratory birds are likely to encounter urban landscapes during migration [33].

During migration, birds need to rest and refuel between bouts of migratory flight [34–36]. Resting and refueling occurs in stopover sites, and, for many migratory birds, the time spent in stopover sites comprises the majority of their migration time [37, 38]. Having access to high quality stopover sites can decrease the time spent on migration [39, 40] and increase the survival and reproductive success of migratory birds [39, 41–43]. When migrating birds encounter urban landscapes, they often use urban green spaces as stopover sites [29, 30, 32, 44]; thus, urban stopover habitat is important for conservation of migratory birds [34, 45–47].

In urban landscapes, breeding birds may use green spaces, areas dominated by impervious surface, or both [48–50]. Urban bird species which do not require green space to nest are generally urban dwellers (e.g., [51, 52]), though some urban utilizers may not need green space for nesting (e.g., [53]). Urban birds that require green space for nesting or foraging during the breeding season are often limited by available habitat, and, therefore, urban green space can provide essential breeding habitat for urban utilizers and even some urban avoiders [54].

Given the importance of urban green space to both migrating and breeding birds, increasing the amount of green space should be a conservation management priority. One approach to increasing green space in urban landscapes is to convert conventional roofs to green roofs. A green roof is a roof that has been covered with a layer of insulation, followed by a water/root barrier, a drainage layer, filter membrane, growing medium (i.e., soil), and, finally, vegetation [55]. Green roofs provide multiple ecological and economic benefits. For example, green roofs provide habitat for arthropods [56], birds [57], and bats [58], aid stormwater management by reducing runoff quantity and pollutant load [59], improve air quality [60], increase roof life [61], decrease urban noise [62], and reduce urban heat island effects [63].

Urban green roofs can host diverse and abundant arthropod communities [56, 64–69]. Green roof arthropod communities can be similar to adjacent ground level habitats [66, 70, 71] but generally have fewer species [64, 72, 73]. Diverse and abundant arthropod communities have the potential to be used by insectivorous birds and other birds which supplement their diet with arthropods as a food source during migration and the breeding season [74–79]. If urban green roofs can provide habitat with increased arthropod food resources, they can increase the quantity and quality of habitat in urban landscapes and may be an effective conservation tool for birds and other wildlife in urban landscapes.

While green roofs have the potential to provide habitat for urban birds, little is known about how birds actually use green roofs (see [57, 80–83]), particularly during migration (but see, [84]). Understanding if migrating and breeding birds are using green roofs in urban landscapes is essential to assess if green roofs can be an effective bird conservation tool [65]. Furthermore, while green roofs might be expected to provide higher quality habitat than conventional roofs, all research to date that compared green and conventional roofs found that birds and their arthropod prey do not use green roofs more than conventional roofs [84, 85]. Comparing arthropod and bird abundance and richness on green versus conventional roofs is necessary for establishing baseline conditions and evaluating the conservation value of green roofs. For regulatory agencies, conservation practitioners, architects, developers, building owners, and building managers, the fundamental question is often whether the resources and effort needed to install a green roof on an existing or new building provides wildlife benefits beyond that provided by conventional roofs and whether the additional cost of installing green roofs can be justified. Knowing how conventional roofs compare to green roofs as wildlife habitat is essential to addressing these questions.

Here, we compare arthropod and bird abundance and richness on green roofs versus comparable nearby conventional roofs in New York City. We use these data to evaluate (1) whether urban green roofs provide better quality habitat than conventional roofs for arthropods and birds, and (2) what species use green roofs versus conventional roofs. Based on existing literature documenting the presence of robust arthropod communities on green roofs, we predict that green roofs host more abundant and rich arthropod and bird communities than conventional roofs. In addition, we predict that birds which utilize arthropods in their diet, such as migratory birds and urban avoiders, will also be more abundant and rich on green roofs. Further, we predict that the composition of bird communities on green roofs will be distinct from bird communities on conventional roofs.

## Field site description

**Fig 1:**
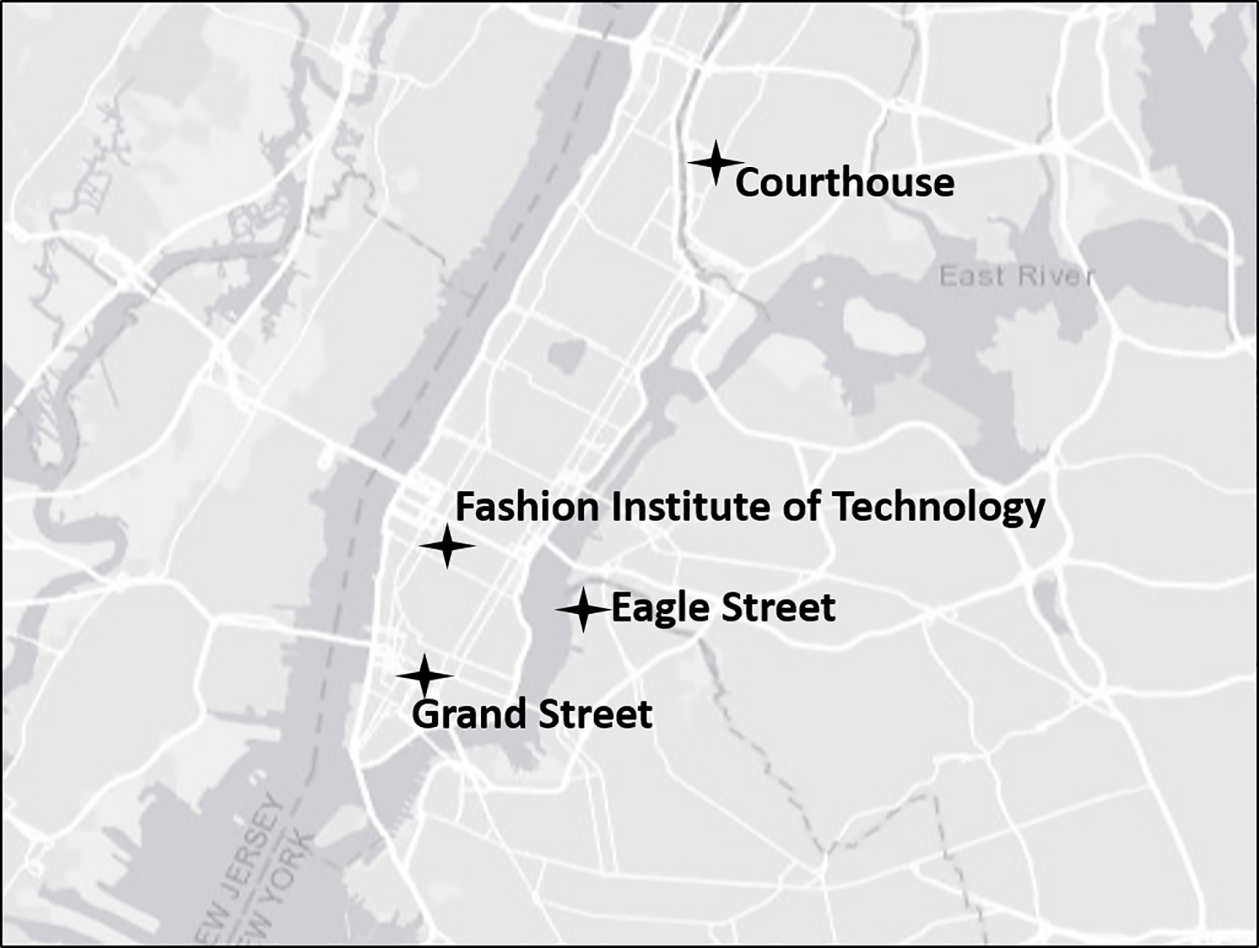
Location of four paired sites (green roofs versus nearby comparable conventional roofs) sampled during spring bird migration (late April and May 2011 and 2012) and the bird breeding season (June to mid July 2011 and 2012) in New York City.

We used four paired sites located throughout New York City for this study (Fig 1); each site had one green roof paired with one conventional roof. Paired roofs shared similar characteristics (e.g., size and elevation) and were located between 40 and 100 m from each other. Sites were located in Little Italy, Manhattan (Grand Street); Chelsea, Manhattan (Fashion Institute of Technology); Greenpoint, Brooklyn (Eagle Street); and downtown Bronx (Courthouse). Roof area ranged from 400 m^2^ to 1,500 m^2^. One of the green roofs was planted entirely with *Sedum* species (low-growing succulents) (Fashion Institute of Technology), one roof was primarily *Sedum* with a thin row of mixed grasses and chives (Courthouse), one roof was a mixed garden with native and non-native plants, shrubs, small trees, and a grass lawn (Grand Street), and one roof contained a farm (Eagle Street). Roof elevation ranged between 2–11 stories, a height range of approximately 11.0 m to 49.5 m. Paired sites did not differ in the amount of surrounding green space within 250 m, 500 m, and 1000 m buffers (χ^2^, P > 0.05). Table 1 contains additional descriptive details for each site.

**Table 1:** Site name, borough, and roof details for four paired sites (green roofs versus nearby comparable conventional roofs) sampled during spring bird migration (late April and May 2011 and 2012) and the bird breeding season (June to mid July 2011 and 2012) in New York City.

Site	Borough	Roof type	Vegetation type	Size (m^2^)	Elevation (m)	% Surrounding green space		
						250m	500m	1000m
Fashion Institute of Technology	Manhattan	Green	Sedum	1500	26.0	4.58	10.74	8.96
		Conventional	n/a	1300	26.0	3.05	9.47	8.56
Courthouse	Bronx	Green	Sedum with a row of mixed grasses and chives	900	49.5	20.79	16.28	20.26
		Conventional	n/a	900	49.5	20.78	15.98	19.65
Eagle Street	Brooklyn	Green	Vegetable farm	500	11.5	11.72	13.69	11.72
		Conventional	n/a	550	11.0	13.27	13.40	11.30
Grand Street	Manhattan	Green	Mixed vegetation: Shrubs, grass lawn, trees, fruits, vegetable, sedum	550	20.5	2.30	6.47	11.88
		Conventional	n/a	400	20.5	1.89	3.98	11.03

Paired sites consisted of one green and one conventional roof located within 100 m of each other. Green and conventional roofs had similar characteristics (size, elevation, and surrounding green space). The percent of green space surrounding each roof was calculated using ArcMap10.2 (ESRI, Redlands, CA). The data source was the 2010 Landcover Raster Dataset, a high resolution (3 ft.^2^) raster available from NYC OpenData (https://data.cityofnewyork.us). Tree canopy cover, grass/shrubs, and bare earth (pervious surface) were classified as green space.

## Methods

Sampling of arthropods and birds occurred from the end of April to the middle of July in 2011 and 2012. The start date each year depended on roof access. We first classified arthropods and bird species based on phenology and further classified bird species by feeding guild and tolerance of urban landscapes. With regard to phenology, we divided our data into two seasonal categories based on bird breeding ecology: spring migration and the breeding season. We designated late April to May 31 as spring migration [86], and June 1 to mid-July the breeding season [87]. In the northern hemisphere, northward bird migration occurs in the spring and southward migration in the fall. In passerine (perching birds) and near passerine bird species, spring migration generally occurs in a shorter amount of time (3–4 weeks) compared to fall migration which takes place over several months [88]. We used spring migration for this study to take advantage of the more compressed time period of migration [88]. Classifying arthropods and bird species by season allowed us to understand the different seasonal wildlife communities on roofs. Seasonal classification of birds also allowed us to identify if birds were using roofs as migratory stopover habitat or as breeding habitat.

We classified bird species into feeding guilds (e.g., insectivore, granivore, frugivore) based on data compiled in the Birds of North America Online [89]. Classifying bird species by feeding guild allowed us to determine if birds were likely preying on the arthropod community also using the roof. We also classified bird species by tolerance of urban landscapes, designating species as urban dwellers, urban utilizers, or urban avoiders [21, 90] based on Johnston [16]. Classifying bird species by their tolerance of urban landscapes allowed us to determine if green roofs are providing habitat for typical urban bird species or if they can provide habitat for species that might otherwise be absent in urban landscapes.

### Arthropod sampling

We used bowl traps to sample rooftop arthropod communities. Bowl traps are effective at capturing a variety of low flying insects, including terrestrial arthropods if set under conditions that allow them to enter the trap, such as when vegetation grows above the lip of the bowl [91]. For our bowl traps, we used white, 591 ml (20 oz) plastic bowls (Georgia-Pacific Consumer Products, Atlanta, GA). We painted the inside of the bowls with fluorescent yellow spray paint (Rustoleum, Vernon Hills, IL) [92] as florescent yellow paint is the most effective color for arthropod bowl traps [93]. The outside of each bowl was not painted and remained white. To account for potential overflow via precipitation, which can occur with untended traps during rain events [64] and lead to the loss of floating specimens, small holes were cut just below the rim of the bowl [91].

Sampling locations on each roof were chosen using a random number generator, and the locations were maintained for the length of the sampling season. Traps were set at a rate of one trap per 100 m^2^. Bowl traps were secured to green roofs by inserting bamboo stakes through the edge of the bowl into the substrate and to conventional roofs by attaching them with Velcro® tape. Bowl traps are typically set with a pre-mixed solution of saltwater and dish detergent. However, to account for the dry, windy conditions which occur on roofs and our ability to visit sites only once per week, propylene glycol was used instead of water. Using propylene glycol in bowl traps prevents rapid desiccation and is even suitable for desert environments [94].

At the end of each weekly sampling period (approximately seven days), the arthropods and solution were removed from the traps. The arthropods were filtered from the trapping solution, moved to labeled glass storage vials, and preserved in 70% ethanol until identification. Using a stereoscopic microscope and Borror & Delong's Introduction to the Study of Insects, 7th Ed. [95], hexapods, isopods, and spiders were identified to order while all other arthropods were identified to subclass or class.

If more than 300 individuals of a species were counted in a single storage vial, that species was sub-sampled, and the total number of individuals was estimated. Sub-sampling was conducted by placing collections of species into a watch glass with a grid drawn onto the bottom [96–98]. The grid consisted of 64 numbered squares each measuring 5 mm x 5 mm. Arthropods were distributed as evenly as possible, and a random number generator was used to select five squares. All individuals in those five squares were counted, and an average number of individuals per square was calculated. The total number of individuals in the watch glass was then estimated based on the average number of arthropods per square with any individuals that fell outside of the grid added to the total number.

To analyze arthropod abundance and richness, we calculated the number of individuals and taxonomic groups per trap, for each roof for every sampling [99]. The number of arthropods per trap was obtained by taking the total number of arthropods collected on a roof and dividing it by the number of traps collected. Richness was used for analysis instead of diversity because this measure gives even weight to rare species/taxonomic groups and is easily interpretable [100]. Using richness also allowed us to more directly compare our results to those of other urban green space studies (e.g., [49, 101, 102]).

### Bird monitoring

Bird presence and relative abundance were determined by recording species-specific vocalizations using automated acoustic recorders (Wildlife Acoustics, Maynard, MA, Model SM2). These acoustic recorders were programmed to record during selected times: from one-half hour before civil sunrise to one-half hour after civil sunset. All vocalizations were recorded to internal memory cards and later transferred to external hard drives.

Sound files were transformed into spectrogram format using Audacity ® (1.3 Beta). A spectrogram is a visual depiction of sound in which the x-axis is time and the y-axis is frequency (hertz, Hz). When a vocalization or irregular noise occurs, the sound is condensed and results in a distinct signature ranging between frequencies.

Song bird vocalizations typically range from 3 to 5 kHz [103], but certain species can have a greater range within a single vocalization. For example, brown-headed cowbird (*Molothrus ater*) vocalizations can range from 0.7 kHz to nearly 11 kHz [104]. Spruce grouse (*Falcipennis canadensis*) vocalize as low as 0.8 kHz, while blackpoll warbler (*Setophaga striata*) vocalizations can reach 9 kHz [105].

Urban landscapes can be noisy and often contain ambient background noise (e.g., traffic) and more intense local noise (e.g., vehicle brakes, sirens, and trains) that can make it difficult to perceive bird vocalizations within recordings. Our recordings in New York City had a consistent low frequency background noise below 1.75 kHz. To help isolate vocalizations in our recordings from this low frequency background noise, we filtered, but did not completely eliminate, frequencies below 1.5 kHz (Rolloff db per octave = 6 db, filter quality = 0.71). This approach potentially impacted identification of low frequency vocalizations, such as those from mourning doves (*Zenaida macroura*), which vocalize between 0.4 and 0.75 kHz [106]. However, identification of this species would be difficult regardless of the filter due to the ambient background noise.

A noise removal filter was then applied to the recording. Noise removal filters reduce constant background noise. Noise removal does not reduce irregular noise such as bird vocalizations or other irregular noises typical of an urban landscape. To remove noise, we created a noise profile extracted from a small region of the file which only contained background noise and no irregular noises. That noise profile was then used to filter background noise from the rest of the file (noise reductions = 24 dB, frequency smoothing = 150 Hz, attack/decay time = 0.15 seconds).

Vocalizations were initially identified to species by visually locating the vocalizations in the spectrogram and then identifying the vocalization to species acoustically. To account for the often continuous singing of certain species (e.g., northern mockingbirds, *Mimus polyglottos*), individual vocalizations were counted as those which were separated by at least five minutes. If vocalizations were simultaneous or obviously from two different individuals, both vocalizations were counted. Vocalizations of house sparrows (*Passer domesticus*) were not included in this analysis; their ubiquity and frequent vocalizations made them difficult to quantify acoustically (e.g., multiple overlapping vocalizations from different birds for up to several hours). Only recordings from the first two hours of each day (one-half hour prior to civil sunrise and one and one-half hours after civil sunrise), the time when most birds peak in vocal activity during the dawn chorus [107], were searched for bird vocalizations.

Recordings from every morning during the designated spring migration season were analyzed, except for days with rain which produces noise interference and prohibits analysis. During the breeding season, when migrants are generally absent, breeding birds are expected to be present at a site on a consistent basis; thus, analysis was conducted on recordings collected once every ten days, a time period that is also used for other breeding bird survey protocols [108, 109]. Bird abundance was analyzed by calculating the number of vocalizations and number of species vocalizing per hour. To further explore the differences between bird community structure we created a rank-abundance curve for green and conventional roofs [110] for both spring migration and the breeding season. A rank abundance curve visualizes the distribution of abundance across species displaying community evenness.

### Statistics

Because green roofs provide more resources for arthropods and birds than conventional roofs, we expected to document a greater abundance and richness of arthropods and birds on green roofs; thus we used one-tailed tests to test our hypotheses, setting alpha at 0.10. Because our abundance and richness data for arthropods and birds did not follow a normal distribution, we used Wilcoxon signed-rank tests. For our paired sample size of n = 4, the lowest possible exact p-value for a one-tailed Wilcoxon signed-rank test is 0.062 [111].

To further visualize differences in the bird communities between green and conventional roofs, we calculated a Bray-Curtis dissimilarity index [110] and used non-metric multidimensional scaling (nMDS) to plot a two-dimensional relationship between roofs using XLSTAT Ecology (Version 19.02).

### Ethics statement

As this study was strictly observational with regards to birds and did not impact their behavior, no approval or waiver was required by Fordham University's IACUC. IACUC approval or waiver is not required for studies of arthropods.

## Results

### Arthropods

#### Spring migration

**Fig 2:**
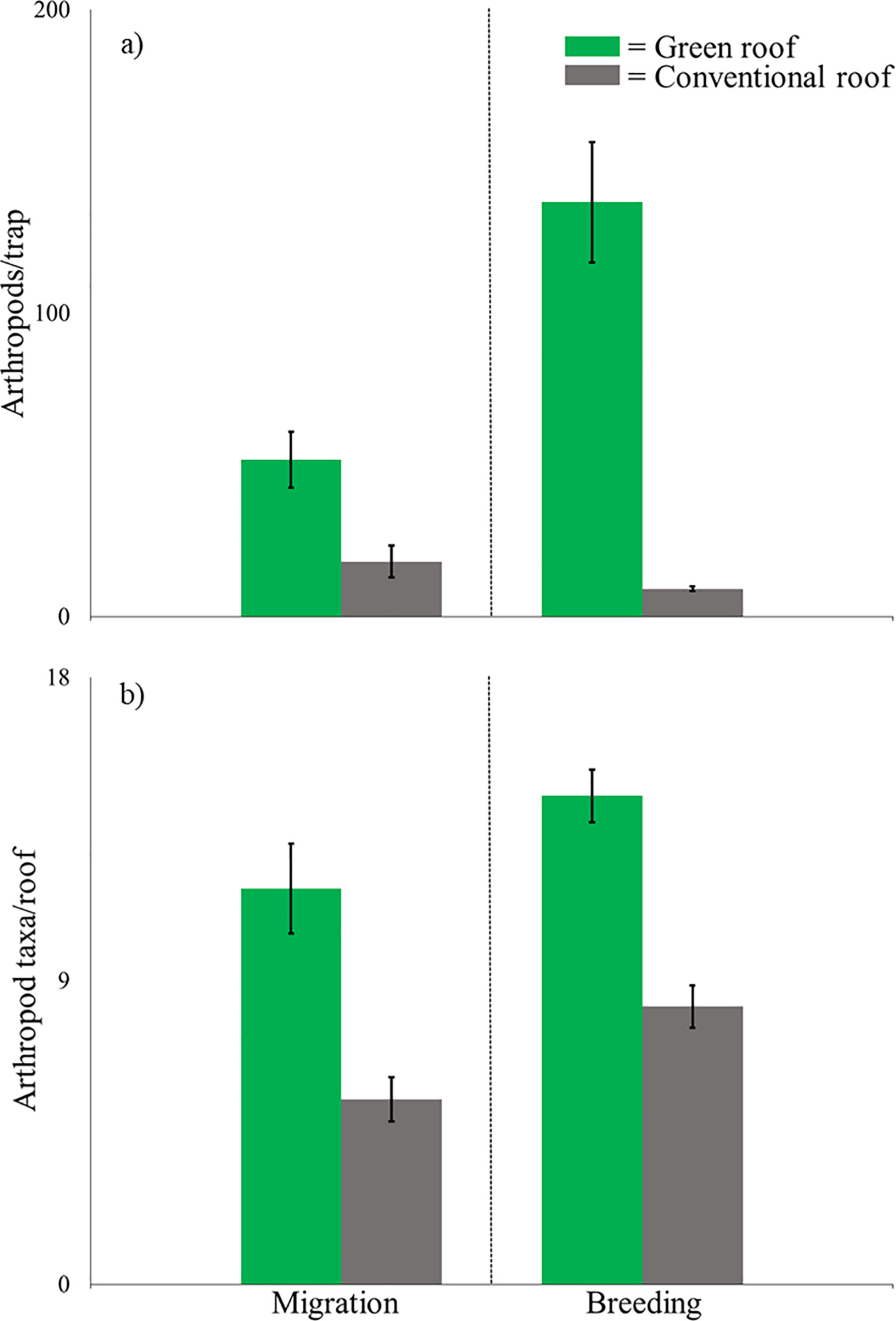
**a) Average arthropods per trap and b) average arthropod taxa per roof on four paired green and conventional roofs during spring bird migration (late April and May 2011 and 2012) and bird breeding season (June to mid July 2011 and 2012) in New York City.** Arthropods were collected using bowl traps. All differences are significant (Wilcoxon signed-rank, P = 0.062).

A total of 13,313 arthropods from 17 taxa were collected during spring migration in two consecutive years on eight roofs (four green and four conventional). 9,939 individuals and 17 taxa were collected on green roofs while 3,374 individuals and nine taxa were collected on conventional roofs (Table 2). Arthropods were more abundant and rich on green roofs than conventional roofs with an average of 51.8 (±18.4 SE) arthropods/trap and 11.75 (±2.7 SE) arthropod taxa/roof collected on green roofs compared to 18.3 (±10.3 SE) arthropods/trap and 5.5 (±1.3 SE) arthropod taxa/roof collected on conventional roofs (P = 0.062, Fig 2). Green roofs were dominated by Diptera (32.1%), Hemiptera (27.2%), Collembolla (16.4%), Thysanoptera (13.2%), Hymenoptera (4.6%), and Aranaea (2.6%), while conventional roofs were dominated by Thysanoptera (50.5%), Hemiptera (34.0%), Diptera (12.2%), and Hymenoptera (2.8%) (Table 2).

**Table 2:** The composition of arthropods collected on four paired green and conventional roofs during spring bird migration (late April and May 2011 and 2012) and the bird breeding season (June to mid July 2011 and 2012) in New York City.

Season	Taxon	Green roofs		Taxon	Conventional roofs	
		Number collected	Percent of total collected		Number collected	Percent of total collected
**Spring Migration**	Diptera	3195	32.15	Thysanoptera	1705	50.53
	Hemiptera	2699	27.16	Hemiptera	1147	34
	Collembola	1629	16.39	Diptera	410	12.15
	Thysanoptera	1315	13.23	Hymenoptera	93	2.76
	Hymenoptera	460	4.63	Coleoptera	8	0.24
	Araneae	252	2.54	Lepidoptera	6	0.18
	Coleoptera	113	1.14	Acari	2	0.06
	Isopoda	104	1.05	Araneae	2	0.06
	Orthoptera	99	1	Neuroptera	1	0.03
	Lepidoptera	30	0.3			
	Gastropoda	15	0.15			
	Acari	14	0.14			
	Lepidoptera larvae	5	0.05			
	Neuroptera	4	0.04			
	Megadrilacea	2	0.02			
	Trichoptera	2	0.02			
	Diplopoda	1	0.01			
Total collected during migration		9939			3374	
**Breeding Season**	Thysanoptera	13022	58.45	Hemiptera	568	39.39
	Diptera	2717	12.20	Diptera	394	27.32
	Collembola	2191	9.84	Thysanoptera	350	24.27
	Hemiptera	1319	5.92	Hymenoptera	53	3.68
	Hymenoptera	1115	5.01	Coleoptera	46	3.19
	Coleoptera	569	2.55	Araneae	12	0.83
	Acari	480	2.15	Lepidoptera	12	0.83
	Araneae	421	1.89	Blatteodea	1	0.07
	Lepidoptera	148	0.66	Diplopoda	1	0.07
	Gastropoda	115	0.52	Isopoda	1	0.07
	Orthoptera	64	0.29	Collembola	1	0.07
	Isopoda	55	0.25	Neuroptera	1	0.07
	Phthiraptera	26	0.12	Phthiraptera	1	0.07
	Lepidoptera larvae	19	0.09	Trichoptera	1	0.07
	UID	6	0.03			
	Dermaptera	5	0.02			
	Diplopoda	2	0.01			
	Neuroptera	2	0.01			
	Thysanura	1	0.00			
Total collected during breeding		22277			1442	** **

Arthropods were collected using yellow bowl traps set at one trap per 100 m^2^. UID indicates individuals that were unable to be identified.

#### Breeding season

A total of 23,719 arthropods of 20 taxa were collected during the breeding season in two consecutive years on eight roofs (four green and four conventional). 22,277 individuals and 18 taxa were collected on green roofs, while 1,442 individuals and 14 taxa were collected on conventional roofs (Table 2). Arthropods were more abundant and rich on green roofs than conventional roofs with an average of 136.7 (±39.7 SE) arthropods/trap and 14.5 (±1.6 SE) arthropod taxa/roof collected on green roofs compared to 9.3 (±1.7 SE) arthropods/trap and 8.25 (±1.3 SE) arthropod taxa/roof collected on conventional roofs (P = 0.062, Fig 2). Green roofs were dominated by Thysanoptera (58.5%), Diptera (12.2%), Collembolla (9.8%), Hemiptera (5.9%), and Hymenoptera (5.0%), while conventional roofs were dominated by Hemiptera (39.4%), Diptera (27.3%), and Thysanoptera (24.3%) (Table 2).

### Birds

#### Spring migration

**Fig 3:**
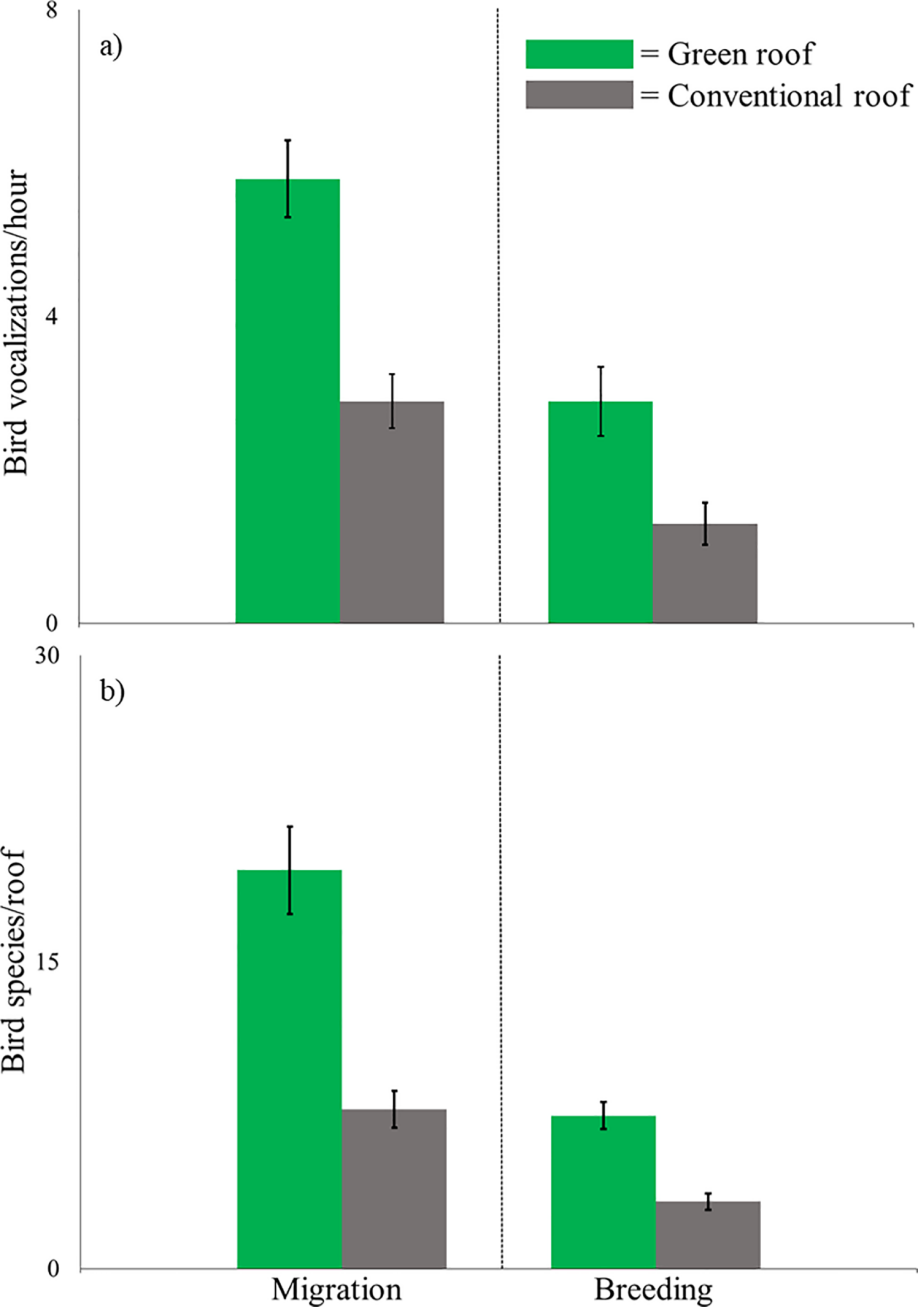
**a) Average bird vocalizations per hour and b) average bird species per roof on four paired green and conventional roofs during spring bird migration (late April and May 2011 and 2012) and the breeding season (June to mid July 2011 and 2012) in New York City.** Bird vocalizations were recorded from one-half hour prior to sunrise to one and one-half hours after sunrise using acoustic recorders. Recordings were then visualized in spectrogram format, and bird vocalizations were identified to species. All differences are significant (Wilcoxon signed-rank, P = 0.062).

**Fig 4:**
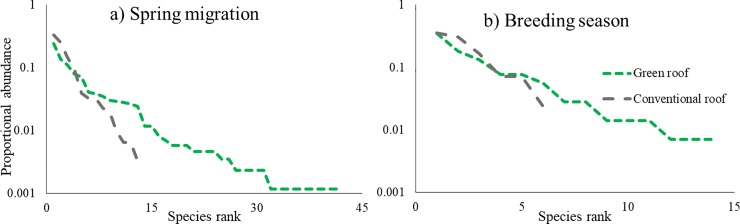
Proportional bird species abundance rank curve for paired green and conventional roofs during spring migration and the breeding season (late April and May 2011 and 2012). Abundance was determined through recordings of vocalizations from one-half hour prior to sunrise to one and one-half hours after sunrise. House sparrows were not included in the analysis.

During spring migration, more bird species were recorded on green roofs (19.50 ±4.3 SE species/roof) than conventional roofs (7.75 ±1.8 SE species/roof) (P = 0.062), and a total of 41 bird species were recorded on green roofs compared to 14 species on conventional roofs (Fig 3). Bird abundance was more evenly distributed across species on green roofs than on conventional roofs (Fig 4). In addition, on an hourly basis, we recorded more vocalizations on green roofs (5.77 ±1.0 SE vocalizations/hour) than conventional roofs (2.91 ±0.7 SE vocalizations/hour) (P = 0.062, Fig 3). The most common vocalizations on both green and conventional roofs were from northern mockingbirds (1.4 vocalizations/hour and 0.69 vocalizations/hour, respectively, Table 3). The next most common vocalizations on green roofs were from, in descending order of frequency: chimney swifts (*Chaetura pelagica*), European starlings (*Sturnus vulgaris*), and herring gulls (*Larus argentatus*). These same species were also the most commonly recorded on conventional roofs, but chimney swift vocalizations were much less common than European starling and herring gull vocalizations. On green roofs, we documented an average of 0.64 vocalizations/hour that could not be identified with confidence, so they were marked as unidentified [112]. All vocalizations on conventional roofs were identified.

**Table 3:** Bird species, diet type, response to urban landscape, and average vocalizations per hour on paired green and conventional roofs during spring bird migration (late April through May 2011 and 2012) in New York City.

Common name	Scientific name	Diet type	Response to urban landscape	Average vocalizations/hour	
				Green	Conventional
Northern mockingbird	*Mimus polyglottos*	Omnivore	Utilizer	1.41	0.70
Chimney swift	*Chaetura pelagica*	Insectivore	Dweller	0.80	0.20
UID*	n/a	n/a	n/a	0.64	
European starling	*Sturnus vulgaris*	Omnivore	Dweller	0.47	0.52
Herring gull	*Larus argentatus*	Omnivore	Utilizer	0.41	0.30
American goldfinch	*Spinus tristis*	Granivore	Avoider	0.24	<0.02
Northern cardinal	*Cardinalis cardinalis*	Granivore	Utilizer	0.22	
Song sparrow	*Melospiza melodia*	Insectivore	Utilizer	0.20	
Laughing gull	*Leucophaeus atricilla*	Omnivore	Utilizer	0.18	0.07
House finch	*Haemorhous mexicanus*	Granivore	Dweller	0.17	0.05
Cedar waxwing	*Bombycilla cedrorum*	Frugivore/Insectivore	Avoider	0.16	0.07
Mourning dove	*Zenaida macroura*	Granivore	Utilizer	0.16	0.08
American robin	*Turdus migratorius*	Insectivore	Utilizer	0.14	<0.02
American kestrel	*Falco sparverius*	Carnivore/Insectivore	Utilizer	0.07	<0.01
Barn swallow	*Hirundo rustica*	Insectivore	Utilizer	0.07	0.02
American crow	*Corvus brachyrhynchos*	Omnivore	Utilizer	0.05	<0.02
Brown-headed cowbird	*Molothrus ater*	Granivore	Avoider	0.04	
Blue jay	*Cyanocitta cristata*	Omnivore	Utilizer	0.03	
Peregrine falcon	*Falco peregrinus*	Carnivore	Utilizer	0.03	
Rock pigeon	*Columba livia*	Granivore	Dweller	0.03	<0.02
Common raven	*Corvus corax*	Omnivore	Utilizer	0.03	
Gray catbird	*Dumetella carolinensis*	Insectivore	Utilizer	0.03	
Ruby-throated hummingbird	*Archilochus colubris*	Nectivore/Insectivore	Avoider	0.03	
White-throated sparrow	*Zonotrichia albicollis*	Granivore/Insectivore	Avoider	0.03	
Canada goose	*Branta Canadensis*	Foliovore	Utilizer	0.02	
Fish crow	*Corvus ossifragus*	Omnivore	Utilizer	0.02	
American redstart	*Setophaga ruticilla*	Insectivore	Utilizer	<0.02	
Baltimore oriole	*Icterus galbula*	Insectivore/Frugivore	Avoider	<0.02	
House wren	*Troglodytes aedon*	Insectivore	Utilizer	<0.02	
Red-winged blackbird	*Agelaius phoeniceus*	Insectivore	Avoider	<0.02	
Tufted titmouse	*Baeolophus bicolor*	Insectivore	Utilizer	<0.02	
American woodcock	*Scolopax minor*	Insectivore	Avoider	<0.01	
Black and white warbler	*Mniotilta varia*	Insectivore	Avoider	<0.01	
Carolina wren	*Thryothorus ludovicianus*	Insectivore	Avoider	<0.01	
Chipping sparrow	*Spizella passerina*	Granivore	Utilizer	<0.01	
Eastern towhee	*Pipilo erythrophthalmus*	Omnivore	Avoider	<0.01	
Field sparrow	*Spizella pusilla*	Insectivore	Avoider	<0.01	
Hermit thrush	*Catharus guttatus*	Insectivore	Avoider	<0.01	
Magnolia warbler	*Setophaga magnolia*	Insectivore	Avoider	<0.01	
Red-tailed hawk	*Buteo jamaicensis*	Carnivore	Utilizer	<0.01	
Willow flycatcher	*Empidonax traillii*	Insectivore	Utilizer	<0.01	
Yellow-rumped warbler	*Setophaga coronata*	Insectivore/Frugivore	Avoider	<0.01	

Acoustic recorders were used to record vocalizations from one-half hour prior to sunrise and one and one-half hours after sunrise. House sparrows were not included in the analysis but were present on both green and conventional roofs. UID indicates vocalizations that were unable to be identified. Blank cells indicate that no vocalizations from that species were recorded.

**Fig 5:**
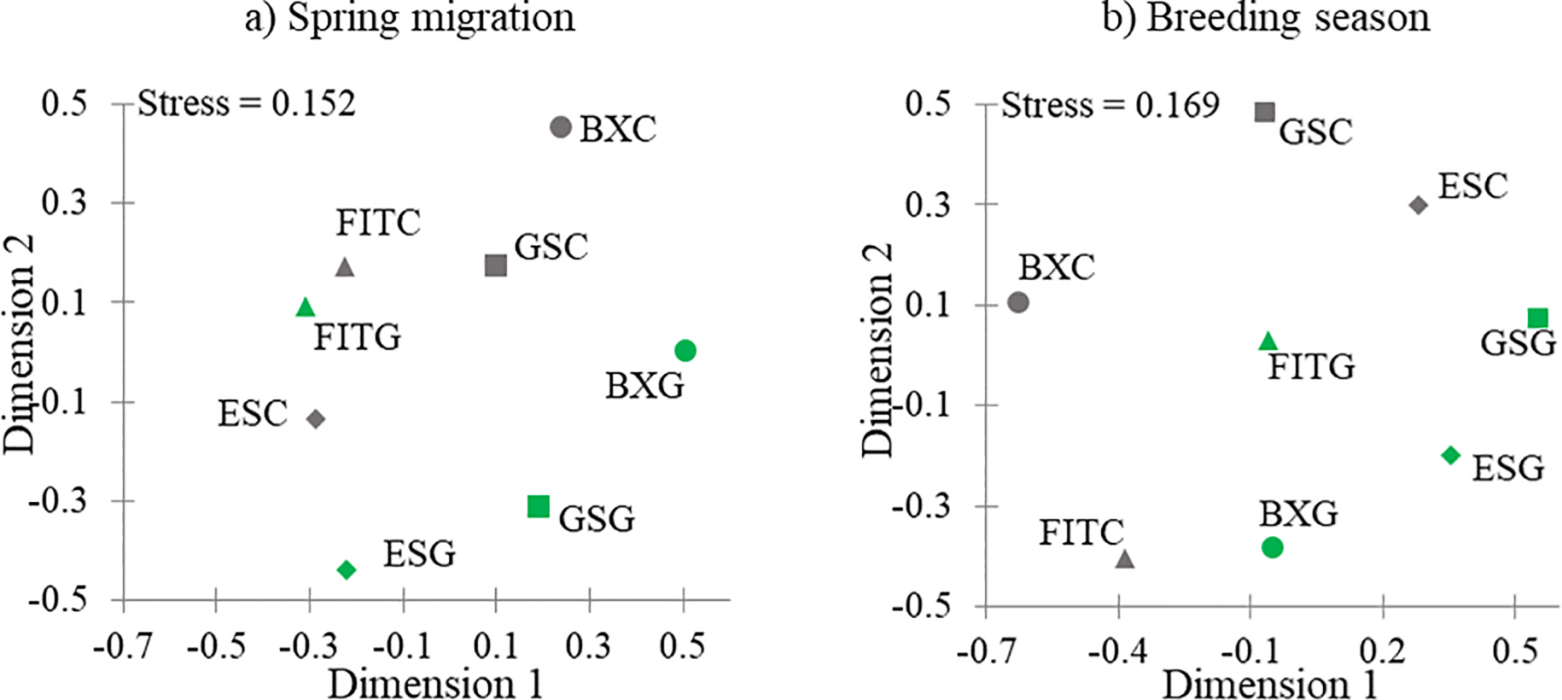
Similarity of bird communities on green and conventional roofs during spring migration (late April through May 2011 and 2012) and the breeding season (June through July 2011 and 2012) in New York City displayed using non-metric multidimensional scaling (nMDS). We created a matrix of dissimilarity between sites using a Bray-Curtis analysis and created a plot displaying similarity using nMDS. Green indicates a green roof; dark grey indicates a conventional roof. FIT = Fashion Institute of Technology, GSC = Grand Street, ES = Eagle Street, BX = Courthouse.

Species composition varied by roof, but two urban avoider species were recorded on conventional roofs: cedar waxwings (*Bombycilla cedrorum*), a frugivore/insectivore, and American goldfinches (*Spinus tristis*), a granivore; however, neither species was recorded on more than one conventional roof. Urban avoider species were more common on green roofs, with 15 urban avoider species recorded (Table 3). Of these 15 urban avoider species on green roofs, seven species were recorded on more than one occasion, and five species occurred on more than one green roof. Conventional roofs were dominated by omnivorous bird species, while most bird species on green roofs were either insectivores or species which supplement their primary diet with arthropods (Table 3). In general, bird community composition on green roofs was more similar to other green roofs than their paired conventional roofs with the exception of the Fashion Institute of Technology green roof which was most similar to its paired conventional roof (Fig 5).

#### Breeding season

During the breeding season, we documented more bird species on green roofs (7.50 ±1.3 SE species/roof) than conventional roofs (3.25 ±0.8 SE species/roof) (P = 0.062), and a total of 14 bird species were recorded on green roofs compared to six species on conventional roofs (Fig 3). In addition, on an hourly basis, we recorded vocalizations from more bird species on green roofs (3.35 ±0.9 SE vocalizations/hour) than conventional roofs (1.23 ±0.6 SE vocalizations/hours) (P = 0.062, Fig 3). Bird abundance was more evenly distributed across species on green roofs than on conventional roofs (Fig 4).

The most common vocalizations on green roofs were from northern mockingbirds (1.25 vocalizations/hour) while European starlings were the most commonly recorded on conventional roofs (0.38 vocalizations/hour, Table 4). The next most common vocalizations on green roofs were from European starlings, herring gulls, chimney swifts, and northern cardinals (*Cardinalis cardinalis*) while, on conventional roofs, vocalizations from herring gulls and northern mockingbirds were next most common. Generally, the breeding bird communities on green roofs were more similar to the bird communities on other green roofs than to their paired conventional roofs (Fig 5).

**Table 4:** Bird species, diet type, response to urban landscape, and average vocalizations per hour on paired green and conventional roofs during the breeding season (June through mid-July 2011 and 2012).

Common name	Scientific name	Diet type	Response to urban landscape	Average vocalizations/hour	
				Green	Conventional
Northern mockingbird	*Mimus polyglottos*	Omnivore	Utilizer	1.25	0.18
European starling	*Sturnus vulgaris*	Omnivore	Dweller	0.65	0.38
Herring gull	*Larus argentatus*	Omnivore	Utilizer	0.48	0.33
Chimney swift	*Chaetura pelagica*	Insectivore	Dweller	0.28	
Northern cardinal	*Cardinalis cardinalis*	Granivore	Utilizer	0.28	0.08
Barn swallow	*Hirundo rustica*	Insectivore	Utilizer	0.20	
American goldfinch	*Spinus tristis*	Granivore	Avoider	0.10	
American robin	*Turdus migratorius*	Insectivore	Utilizer	0.10	
Mourning dove	*Zenaida macroura*	Granivore	Utilizer	0.05	0.03
American crow	*Corvus brachyrhynchos*	Omnivore	Utilizer	0.05	
Common grackle	*Quiscalus quiscula*	Omnivore	Utilizer	0.05	
UID*	n/a	n/a	n/a	0.03	
American kestrel	*Falco sparverius*	Carnivore/Insectivore	Utilizer	0.03	
Red-winged blackbird	*Agelaius phoeniceus*	Insectivore	Avoider	0.03	
Killdeer	*Charadrius vociferus*	Insectivore	Utilizer	0.03	
Rock pigeon	*Columba livia*	Granivore	Dweller		0.08

Acoustic recorders were used to record vocalizations from one-half hour prior to sunrise one and one-half hours after sunrise. House sparrows were not included in the analysis but were present on both green and conventional roofs. UID indicates vocalizations that were unable to be identified. Blank cells indicate that no vocalizations from that species were recorded.

We recorded two species on green roofs during the breeding season that we did not record during spring migration: common grackles (*Quiscalus quiscula*) and killdeer (*Charadrius vociferous*). We also recorded two urban avoiders on green roofs during the breeding season: American goldfinches and red-winged blackbirds (*Agelaius phoeniceus*); however, we recorded no urban avoiders on conventional roofs during the breeding season.

## Discussion

Continued urbanization will cause disruptive change to invertebrate and vertebrate populations [7–11], and novel approaches to conservation should be considered to offset the negative impacts of urbanization [113]. Green roof installation provides a mechanism for increasing green space in urban landscapes and can be an effective tool for wildlife conservation. Urban green roofs can increase connectivity between habitats in urban landscapes [66, 70] and can themselves provide usable wildlife habitat [57]. The benefit of green roofs to birds is, in part, because green roofs can provide habitat for arthropods which are an important element of quality habitat for most birds [74–78]. Our study demonstrates the higher value of green roofs as arthropod habitat compared to conventional roofs. While conventional roofs can provide arthropod habitat when vegetation colonizes portions of the roof [114–116], we show that green roofs host a higher abundance and richness of arthropods.

Birds visiting green roofs can take advantage of the increased arthropod food supply on green roofs, and, thus, green roofs likely provide higher quality habitat than conventional roofs. The increased abundance of arthropods on green roofs may partially explain why the majority of birds we found on green roofs during migration were insectivores, many of which were also urban avoider species, while insectivores were uncommon on conventional roofs. Insectivores were also present on green roofs during the breeding season but not on conventional roofs. Conventional roofs were dominated by omnivorous urban dweller and urban utilizer species, bird species that do not rely on arthropods when foraging [16, 19, 22, 28].

We found fewer urban avoiders on green roofs than urban utilizers. The relative absence of urban avoiders on green roofs may be due to insufficient green roof size or insufficient nearby ground-level green space [16, 19]. The two most common urban avoider species documented during migration, American goldfinches and cedar waxwings [16], were most common at the Courthouse study site, the green roof in our study with the greatest amount of surrounding green space. These two species were also recorded on the Courthouse conventional roof, but on no other conventional roofs. The isolation, diversity, and size of urban parks and gardens influence the richness and abundance of wildlife in such green spaces [23, 27, 117–120], and the same is likely true for urban green roofs.

The number of bird species we recorded on green roofs during migration was higher than expected based on earlier studies in temperate environments [80, 83, 84] (but see [57] for results from a tropical environment). Prior to our research, the only study to describe migratory bird use of green roofs examined green roofs at the Chicago O’Hare Airport [84], a large airport with grass fields and buildings covered by both green and conventional roofs. In contrast to Washburn et al. [84], we found that more birds and more bird species used green roofs during spring migration than during the breeding season. This contrast with our results could be due to differences in surrounding habitat (i.e., an airport versus an urban landscape); however, a difference in when we sampled may also help explain differences in our results. Washburn et al. [84] considered March to be the migration season and recorded greater richness during the breeding season, which they considered to be April through July. The Washburn et al. [84] study presumably considered March to constitute the spring migration season due to the presence of birds such as killdeer which migrate in March [121–123]. However, we considered April and May to be the migration season since most migratory landbirds migrate in April and May rather than March, with the exception of raptor species or inland shorebirds such as killdeer [124, 125].

Migrating birds that pass through urban landscapes can use urban green space to adequately refill fat stores during stopovers [29, 126], and the birds we recorded during spring migration were likely using urban green roofs as stopover habitat. Migrating birds will move short distances to more suitable habitats shortly after landing in a stopover habitat [35, 127, 128], and migrating birds using urban green space as stopover habitat demonstrate high levels of movement within the first day of arriving [44, 129]. While we recorded migrating birds stopping over on green roofs, the abundance of such birds was limited, which could indicate that green roofs are insufficient habitat for prolonged stopovers [130]. Another interpretation of this limited abundance of migrating birds on our green roofs could also indicate that green roofs are the only stopover habitat available to migrants [131] or that green roofs are being used as temporary habitats between nocturnal flights [132].

Ground nesting birds are known to nest on both green roofs [83, 84] and on conventional roofs [133, 134]. However, many green roofs are composed of sedum or other low-lying plants and may lack sufficient vegetative structure to constitute useable nesting habitat for other bird species. We observed northern mockingbirds, a tree and shrub nesting species [135], nesting on green roofs during our study, but we found no birds nesting on conventional roofs.

Comparing the composition of the bird communities on green versus conventional roofs showed that bird communities on green roofs are generally more similar to bird communities on other green roofs than to their paired conventional roofs during both spring migration and the breeding season (Fig 5). The Fashion Institute of Technology site was the exception, with the green and conventional roofs being more similar to each other during spring migration. The Fashion Institute of Technology green roof is composed entirely of *Sedum* and is fairly isolated from surrounding green space, and both factors likely influence green roof bird communities (Table 1).

The composition of vegetation on green roofs can vary widely, from simple monocultures of sedum species to farms to highly mixed vegetative communities that include forbs, grasses, shrubs, and even trees. How variation in green roof vegetative communities impacts local bird communities is not well understood. Though our sample size of green roofs was small, our results suggest that green roofs act as unique and valuable habitat when compared to nearby conventional roofs. However, similar to arthropod communities on green roofs [136], the composition of bird communities on green roofs is likely influenced by green roof size, vegetation diversity, and distance to other nearby green spaces. The influence of roof size, vegetation diversity, isolation, and elevation on bird abundance and diversity should be studied to more fully understand how green roofs can be used for bird conservation.

Most green roofs could be improved to increase their value to both arthropods and birds. For example, deeper substrate, increased vegetative diversity, and planting with native plants may benefit native fauna. We often observed birds using any available elevated platform as a perch (e.g., HVAC units, antennae, and weather stations), and installing bird perches within the vegetated areas of green roofs would likely benefit birds and increase bird use of green roofs. Planners should also consider connectivity to nearby green spaces [66, 70]. Finally, green roof planners and installers should attempt to keep green roofs safe for birds by using bird-friendly glass and planting vegetation in ways that reduce potential bird-window collisions.

Bird abundance and richness in our study was higher than that found in similar studies in other temperate environments (e.g., [80, 84]). Even so, our bird abundance and richness numbers are likely an underestimate. We used acoustic recordings to document abundance and richness, and acoustic recordings are unlikely to record every bird present on a roof given that birds do not always vocalize. In addition, filtering sound files to reduce background noise may have resulted in lost low frequency vocalizations, and the remaining background noise may have masked other vocalizations. Acoustic recorders are useful for long-term monitoring or for monitoring in locations that are difficult to access regularly [137–139], such as green roofs. However, because of substantial and chronic background noise, using acoustic recorders in urban landscapes likely further reduces their effectiveness. Had we surveyed in person for the same amount of time as we deployed acoustic recorders, we likely would have documented more birds and more bird species [112, 138, 140].

The creation of new green space in urban landscapes increases the amount of habitat available to birds. While such green space can also expose birds to increased anthropogenic threats [141, 142], these threats are often offset by the benefits of additional habitat which supports bird populations [31, 79, 143]. Consequently, creating urban green space remains important for bird conservation [120, 144]. Green roofs generally constitute higher quality arthropod and bird habitat than conventional roofs and can provide valuable habitat in landscapes previously devoid of vegetation. Our research demonstrates that urban green roofs provide greater conservation benefits compared to conventional roofs: green roofs host more abundant and rich arthropod and bird communities, host more urban utilizer and urban avoider bird species, and provide important habitat for both migrating and breeding birds. Green roof installation is a sustainable way to increase urban green space and should be considered as an important tool for migrating and breeding bird conservation in urban landscapes.

## Supporting information

S1 Fig Grand Street study site: One of four paired study sites used to survey arthropods and birds in 2011 and 2012. These photos show both the green roof (left) and the conventional roof (right). Paired study sites were surveyed for arthropods and birds during spring bird migration (late April and May 2011 and 2012) and the bird breeding season (June to mid July 2011 and 2012) in New York City to compare wildlife use of green roofs with nearby comparable conventional roofs.
(TIF)
