# Supplementary figures and images for: Urban green roofs provide habitat for migrating and breeding birds and their arthropod prey
Source: PLoS One. 2018 Aug 29;13(8):e0202298. doi: 10.1371/journal.pone.0202298 (PMC6114707; doi:10.1371/journal.pone.0202298)

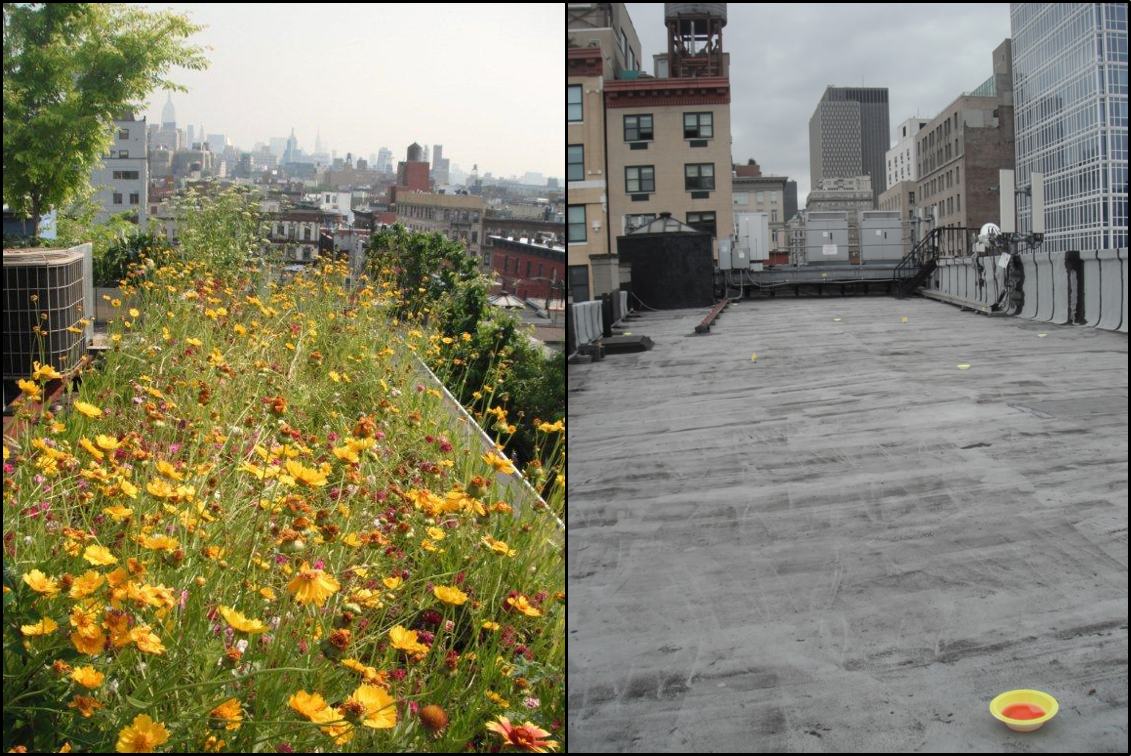

Supplement: S1 Fig — These photos show both the green roof (left) and the conventional roof (right). Paired study sites were surveyed for arthropods and birds during spring bird migration (late April and May 2011 and 2012) and the bird breeding season (June to mid July 2011 and 2012) in New York City to compare wildlife use of green roofs with nearby comparable conventional roofs. (TIF) [file pone.0202298.s001.tif]
